# External validation of inpatient neonatal mortality prediction models in high-mortality settings

**DOI:** 10.1186/s12916-022-02439-5

**Published:** 2022-08-03

**Authors:** Timothy Tuti, Gary Collins, Mike English, on behalf of the Clinical Information Network, George Mbevi, John Wainaina, Livingstone Mumelo, Edith Gicheha, Naomi Muinga, Muthoni Ogola, Laura Oyiengo, Caroline Mwangi, Fred Were, Juma Vitalis, Nyumbile Bonface, Roselyne Malangachi, Christine Manyasi, Catherine Mutinda, David Kibiwott Kimutai, Rukia Aden, Caren Emadau, Elizabeth Atieno Jowi, Cecilia Muithya, Charles Nzioki, Supa Tunje, Penina Musyoka, Wagura Mwangi, Agnes Mithamo, Magdalene Kuria, Esther Njiru, Mwangi Ngina, Penina Mwangi, Rachel Inginia, Melab Musabi, Emma Namulala, Grace Ochieng, Lydia Thuranira, Felicitas Makokha, Josephine Ojigo, Beth Maina, Catherine Mutinda, Mary Waiyego, Bernadette Lusweti, Angeline Ithondeka, Julie Barasa, Meshack Liru, Elizabeth Kibaru, Alice Nkirote Nyaribari, Joyce Akuka, Joyce Wangari, Amilia Ngoda, Aggrey Nzavaye Emenwa, Dolphine Mochache, Patricia Nafula Wesakania, George Lipesa, Jane Mbungu, Marystella Mutenyo, Joyce Mbogho, Joan Baswetty, Ann Jambi, Josephine Aritho, Beatrice Njambi, Felisters Mucheke, Zainab Kioni, Lucy Kinyua, Margaret Kethi, Alice Oguda, Salome Nashimiyu Situma, Nancy Gachaja, Loise N. Mwangi, Ruth Mwai, Irginia Wangari Muruga, Nancy Mburu, Celestine Muteshi, Abigael Bwire, Salome Okisa Muyale, Naomi Situma, Faith Mueni, Hellen Mwaura, Rosemary Mututa, Caroline Lavu, Joyce Oketch, Jane Hore Olum, Orina Nyakina, Faith Njeru, Rebecca Chelimo, Margaret Wanjiku Mwaura, Ann Wambugu, Epharus Njeri Mburu, Linda Awino Tindi, Jane Akumu, Ruth Otieno, Slessor Osok, Seline Kulubi, Susan Wanjala, Pauline Njeru, Rebbecca Mukami Mbogo, John Ollongo, Samuel Soita, Judith Mirenja, Mary Nguri, Margaret Waweru, Mary Akoth Oruko, Jeska Kuya, Caroline Muthuri, Esther Muthiani, Esther Mwangi, Joseph Nganga, Benjamin Tanui, Alfred Wanjau, Judith Onsongo, Peter Muigai, Arnest Namayi, Elizabeth Kosiom, Dorcas Cherop, Faith Marete, Johanness Simiyu, Collince Danga, Arthur Otieno Oyugi, Fredrick Keya Okoth, Jalemba Aluvaala

**Affiliations:** 1 KEMRI-Wellcome Trust Research Programme, P.O. Box 43640, Nairobi, Kenya; 2 Centre for Statistics in Medicine, Nuffield Department of Orthopaedics, Rheumatology & Musculoskeletal Sciences, University of Oxford, Oxford, UK; 3 NIHR Oxford Biomedical Research Centre, John Radcliffe Hospital, Oxford, UK; 4 Health Systems Collaborative, Nuffield Department of Medicine, University of Oxford, Oxford, UK; 5 Department of Paediatrics and Child Health, University of Nairobi, Nairobi, Kenya

**Keywords:** Prognosis, Risk factors, Newborn, Hospital mortality, Africa

## Abstract

**Background:**

Two neonatal mortality prediction models, the Neonatal Essential Treatment Score (NETS) which uses treatments prescribed at admission and the Score for Essential Neonatal Symptoms and Signs (SENSS) which uses basic clinical signs, were derived in high-mortality, low-resource settings to utilise data more likely to be available in these settings. In this study, we evaluate the predictive accuracy of two neonatal prediction models for all-cause in-hospital mortality.

**Methods:**

We used retrospectively collected routine clinical data recorded by duty clinicians at admission from 16 Kenyan hospitals used to externally validate and update the SENSS and NETS models that were initially developed from the data from the largest Kenyan maternity hospital to predict in-hospital mortality. Model performance was evaluated by assessing discrimination and calibration. Discrimination, the ability of the model to differentiate between those with and without the outcome, was measured using the c-statistic. Calibration, the agreement between predictions from the model and what was observed, was measured using the calibration intercept and slope (with values of 0 and 1 denoting perfect calibration).

**Results:**

At initial external validation, the estimated mortality risks from the original SENSS and NETS models were markedly overestimated with calibration intercepts of − 0.703 (95% CI − 0.738 to − 0.669) and − 1.109 (95% CI − 1.148 to − 1.069) and too extreme with calibration slopes of 0.565 (95% CI 0.552 to 0.577) and 0.466 (95% CI 0.451 to 0.480), respectively. After model updating, the calibration of the model improved. The updated SENSS and NETS models had calibration intercepts of 0.311 (95% CI 0.282 to 0.350) and 0.032 (95% CI − 0.002 to 0.066) and calibration slopes of 1.029 (95% CI 1.006 to 1.051) and 0.799 (95% CI 0.774 to 0.823), respectively, while showing good discrimination with c-statistics of 0.834 (95% CI 0.829 to 0.839) and 0.775 (95% CI 0.768 to 0.782), respectively. The overall calibration performance of the updated SENSS and NETS models was better than any existing neonatal in-hospital mortality prediction models externally validated for settings comparable to Kenya.

**Conclusion:**

Few prediction models undergo rigorous external validation. We show how external validation using data from multiple locations enables model updating and improving their performance and potential value. The improved models indicate it is possible to predict in-hospital mortality using either treatments or signs and symptoms derived from routine neonatal data from low-resource hospital settings also making possible their use for case-mix adjustment when contrasting similar hospital settings.

**Supplementary Information:**

The online version contains supplementary material available at 10.1186/s12916-022-02439-5.

## Background

Low- and middle-income countries (LMICs) accounted for 98% of the global neonatal mortality in 2018 [1]. Improved delivery of essential interventions in LMICs hospitals can advance the attainment of the Sustainable Development Goal of lowering the neonatal mortality rate considerably [2, 3]. A better understanding of hospitals’ neonatal mortality coupled with consistent and appropriate information on how this mortality varies may enhance efforts to improve hospital care at scale [4, 5]. Without adjustment for patient case-mix efforts to contrast neonatal in-hospital mortality may be misleading because they fail to adjust for neonatal population characteristics [6].

Well-performing prediction models can support better clinical decision-making and case-mix adjustment and subsequently help improve service delivery at the health system level [7]. However, a review of existing neonatal prediction models found them to be ill-suited for routine practice in LMICs due to over-relying on physiological patient measures and treatments that are usually unavailable in LMICs [8]. Such limitations can be addressed by using predictors that are available in LMIC settings and applying recommended approaches to prediction model development and validation [8, 9]. Candidate predictors encompass essential interventions included in the clinical practice guidelines for in-hospital neonatal care developed by the WHO [10] and simple clinical symptoms and signs recommended for assessing illness in these populations in LMIC settings, such data are more easily collectable [11]. Other prediction models for LMICs (e.g. Neonatal Mortality Rate (NMR)-2000 [12]) proposed for sub-Saharan Africa (SSA) still depend on technologies like pulse oximeters which may not be available or routinely used in most hospital settings in SSA [12]. Such models may also show only modest calibration to patient populations in LMICs and are typically developed from relatively small patient cohorts [12]. Even simpler approaches to mortality risk estimation suitable for the LMIC context may therefore be useful.

Prior work suggested neonatal data on essential clinical signs, symptoms, and treatments routinely collected in a low-resource clinical setting might accurately predict in-hospital mortality [13]. Two prediction models were developed in Kenya: (1) the Neonatal Essential Treatment Score (NETS) from treatments prescribed at the time of admission and (2) the Score for Essential Neonatal Symptoms and Signs (SENSS) from basic clinical signs. Both demonstrated reasonably good discrimination performance with AUCROC of 0.89 (95% CI 0.86 to 0.92) and 0.89 (95% CI 0.84 to 0.93), respectively, with their development described in detail elsewhere [13].

However, they were developed using data from a single hospital, and thus, further external validation of these models is recommended to ensure that the risk estimates they produce are reliable and is warranted to support their wider use to help understand and examine the performance variation across hospitals after case-mix adjustment [13–15]. The most valuable characteristic of a prediction model performance is its generalisability to an external population. Differences in the case-mix and outcomes from the population contributing to the development data may influence the calibration of the models [16]. Our objectives therefore were to:

1. Conduct external validation of the NETS and the SENSS models for predicting in-hospital neonatal mortality using routine clinical data from different hospitals
2. Evaluate whether their performance (and generalisability) could be improved by re-estimating the original regression coefficients with updated data from the derivation hospital

## Methods

### Ethics and reporting

The reporting of this study follows the Transparent Reporting of a multivariable prediction model for Individual Prognosis Or Diagnosis (TRIPOD) guidelines, which is a set of recommendations for the reporting of studies developing, validating, or updating prediction models for prognostic purposes [17]. The Scientific and Ethics Review Unit of the Kenya Medical Research Institute (KEMRI) approved the collection of the de-identified data that provides the basis for this study as part of the Clinical Information Network (CIN). The CIN is run in partnership with the Ministry of Health (MoH) and participating hospitals. Individual consent for access to the de-identified patient data was not required.

### Study design and participants

**Fig. 1:**
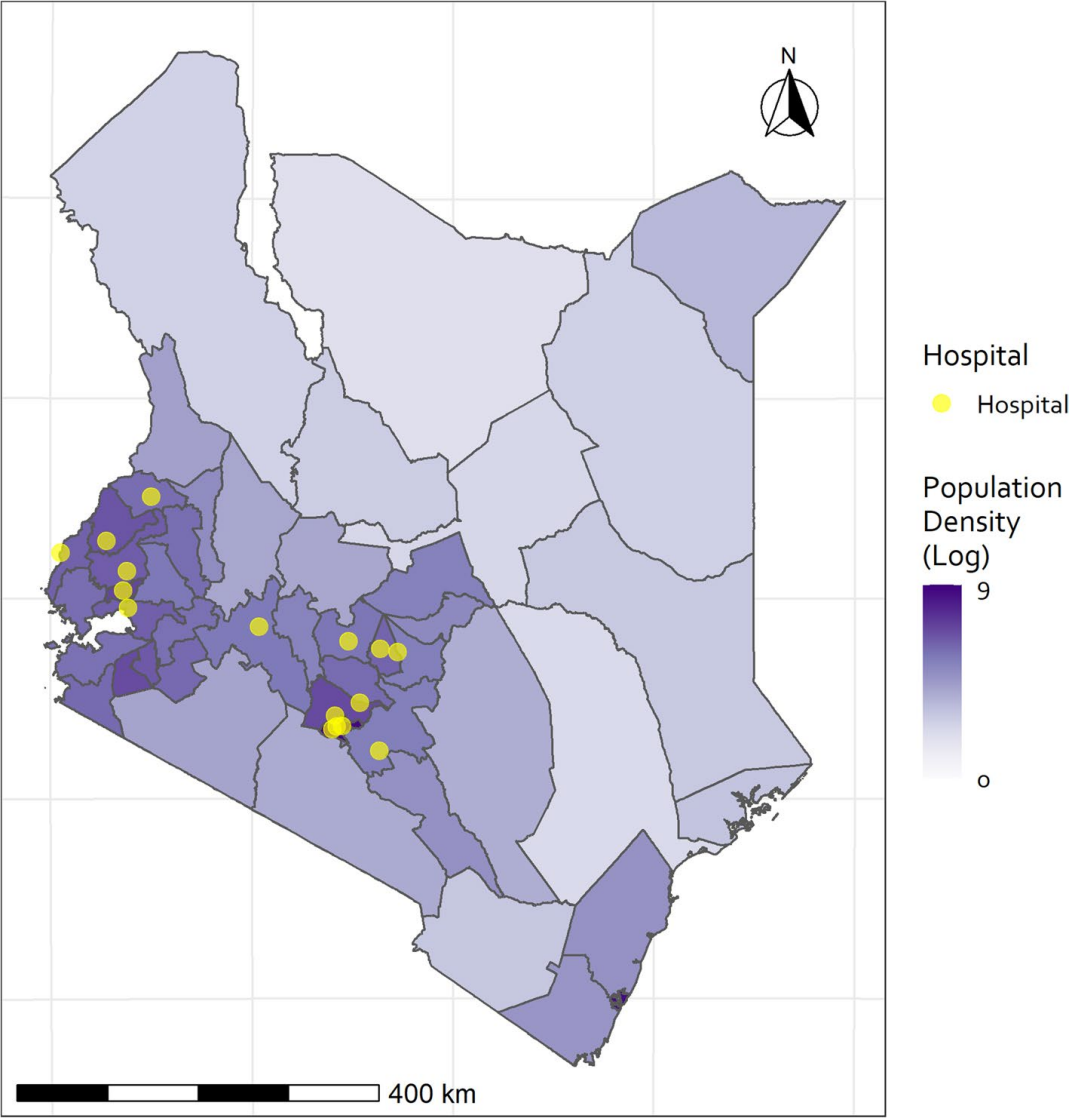
Hospitals providing data for model derivation and external validation represented by the dots. The hospitals that cluster west of the map are in moderate to high malaria transmission zone while the cluster at the centre of the map is in moderate to low malaria transmission zones

The study used data on all patients admitted to the New-Born Units (NBUs) from 16 public hospitals representative of different malaria transmission zones in Kenya, purposefully selected in partnership with the MoH. From the map in Fig. 1, the hospitals that cluster west of the map are in moderate to high malaria transmission zone while the cluster at the centre of the map are in moderate to low malaria transmission zones. These hospitals largely provide maternal care services to immediately surrounding populations including accepting referrals from smaller rural clinics. They were purposefully selected to have moderately sized NBUs with an interquartile range of annual NBU inpatient admissions of 550 to 1640 (Fig. 1).

De-identified patient-level data were obtained after being recorded by clinicians as part of routine care. This data collection system linked to the CIN includes data quality assurance procedures and is described in detail elsewhere [11, 18, 19]. In brief, structured paper newborn admission record (NAR) and NBU exit forms that are endorsed by the Kenyan MoH are the primary data sources for the CIN. CIN supports one data clerk in each hospital to abstract data from the paper hospital records each day for all patients after discharge with the data entered directly into a non-proprietary Research Electronic Data Capture (REDCap) tool [20] with inbuilt range and validity checks. Data entry is guided by a standard operating procedure manual that forms the basis of the data clerks’ training with automated error-checking systems. To ensure no record is missed, the research team benchmarks the admission numbers entered in the CIN database with the aggregate statistics submitted to the MoH. External data quality assurance is done by KEMRI research assistants who perform an on-site concordance check every 3 months by comparing results from 5% randomly selected records they re-enter into REDCap to data clerks’ entries. The overall concordance of the external data quality audits has been ranging between 87 and 92% over time with feedback given to the data clerks and any challenges addressed for continuous improvement of data quality.

This study included neonates admitted to the NBUs between August 2016 and March 2020, from 16 hospitals representing different regions of the country, with 15 hospitals providing the external validation dataset (*n* = 53,909) (Fig. 1) and the 16th hospital dataset used for the model derivation and temporal validation. For objective 2, the data that was used for the model updating (i.e. re-estimating all the original SENSS and NETS regression coefficients) consisted of derivation stage dataset (April 2014 to December 2015: *n* = 5427), temporal validation stage dataset (January 2016 to July 2016: *n* = 1627), and additional data collected from August 2016 to December 2020 (*n* = 8848), all from the same hospital (16th hospital). Model updating is typically required where there is observed deterioration in model performance in the new population (e.g. during model external validation) [21]. We provide explanations of the meaning and significance of the different datasets in Additional file 1: Table S1.

### Outcome

The outcome was all-cause in-hospital neonatal unit mortality. Outcome assessment was blind to predictor distribution as the hospital data clerks were unaware of the study [9].

### Predictors

No new predictors were considered for SENSS and NETS models’ external validation and updating, only those used in the derivation and temporal validation study were included [13, 21]. For the NETS model, the use/non-use of supplementary oxygen, enteral feeds, intravenous fluids, first-line intravenous antibiotics (penicillin and gentamicin), and parenteral phenobarbital predictors were used [10]. For the SENSS model, the presence or absence of difficulty feeding, convulsions, indrawing, central cyanosis, and floppy/inability to suck, as assessed at admission, were used [10, 13]. Neonate’s birth weight by category (< 1 kg, 1.0 to < 1.5 kg, 1.5 to < 2.5 kg, 2.5 to 4.0 kg, and > 4 kg) and sex were also included in both models. Weight was treated as a categorical predictor rather than being continuous, despite categorisation likely causing information loss, based on a priori clinical consensus [9, 10]. Detailed descriptions and arguments for the selection of these variables are provided in the derivation study [13] and in Additional file 1: Table S2 and Additional file 1: Table S3. The proportion of predictor missingness is consistent with previous work in Kenyan hospitals [22].

### Sample size

#### Sample size for model validation

Sample size guidance for external validation of prediction models suggests a minimum recommended 100 events and 100 non-events for validation studies [23]. For SENSS and NETS models, there were 7486/53,909 (13.89%) and 6482/45,090 (14.38%) events (deaths), respectively, with 46,358/53,909 (85.99%) and 38,576/45,090 (85.55%) non-events (survived), respectively.

#### Sample size for model updating

Based on an outcome prevalence of 508/5427 (9.36%) and 447/4840 (9.24%) for the SENSS and NETS derivation datasets, respectively; 10 predictor parameters; and R-squared values of 0.453 and 0.380, using the *pmsampsize* library in R, the required sample sizes required for SENSS and NETS model updating were 323 and 341 patients with 31 and 32 deaths, respectively [24]. There were 7486 (from 53,909 patients) and 6482 deaths (from 45,090 patients) observed for SENSS and NETS models, respectively, which exceeds the required sample sizes [24, 25].

### Missing data

Predictor missingness in the SENSS external validation dataset (Additional file 1: Table S4) ranged from 1.19% (sex) to 14.63% (floppy/inability to suck). The derivation model assumed a missing at random (MAR) mechanism for the observed missingness and performed multiple imputation using the chained equation (MICE) approach [26]. Therefore, for external validation before updating, the same mechanism was assumed. Similar to the derivation study, *mode of delivery*, *outborn*, *Apgar score at 5 min, HIV exposure*, and *outcome* were used as auxiliary variables in the imputation process [13, 27].

Consistent with the NETS model derivation approach, 8819 (16.36%) observations in the external dataset with missing treatment sheets in the patient files were excluded, leaving 45,090 observations with 6482 (14.38%) in-hospital deaths. Multiple imputation was considered inappropriate and therefore not done for NETS where the entire treatment sheets were missing (i.e. no information on any of the treatment predictors was available) because individual missing treatment data was judged to be systematically missing due to the factors not reflected in the dataset. Therefore, all patients with no treatment sheets (8819/53,909 in the external dataset and 2238/8848 in the model updating dataset) and those missing data in any treatment variable in the resultant NETS dataset (9440/45,090 in the NETS external dataset and 941/6610 in the NETS model updating dataset) were dropped from NETS model analyses (Additional file 1: Table S5). Consequently, NETS analyses were complete case analyses based on the missingness of the patient’s sex and birth weight.

### Statistical analysis methods

The overall recommended process of predictive modelling is well articulated in the scientific literature [9, 14, 17, 24, 28]. To externally validate the performance of the original SENSS and NETS models, these models were applied to the CIN dataset from 15 hospitals (geographical external validation). For external validation before updating (i.e. objective one), external validation was done by applying the model coefficients obtained at the model derivation stage to the external validation data [13]. The models and coefficients are presented in Table 1. The SENSS model was fit on each of the 33 imputed datasets (based on 33% of observations missing at least one variable [29]) with parameter estimates combined using Rubin’s rule [30].

**Table 1:** Logistic regression models for NETS and SENSS from derivation study

SENSS:
Linear predictor (*LP*_SENSS_) = − 3.8583 + 5.7580 * ELBW + 3.7082 * VLBW + 0.9232 * LBW − 0.4918 * macrosomia − 0.1336 * Male + 1.3596 * difficulty feeding + 1.3977 * convulsion + 1.9790 * indrawing + 0.9584 * cyanosis + 1.6266 * floppy unable to suck
NETS:
Linear predicator (*LP*_NETS_) = − 4.1521 + 5.6836 * ELBW + 4.5359 * VLBW + 1.4186 * LBW − 0.2927 * macrosomia − 0.3125 * male + 1.3695 * antibiotics + 1.3256 * fluids − 1.9135 * feeds + 0.6142 * oxygen + 2.5947 * phenobarbital

For each variable, the presence of the indicator takes a value of 1, and the absence takes a value of 0. The coefficients are summated to give the linear predictor, which is then converted to the predicted probability of in-hospital mortality [13]

*ELBW* Extremely low birth weight, *LBW* Low birth weight, *LP* Linear predictor, *NETS* Neonatal Essential Treatment Score, *SENSS* Score of Essential Neonatal Symptoms and Signs, *VLBW* Very low birth weight

Model calibration was assessed by both plotting the predicted probability of in-hospital death against the observed proportion and calculating the calibration slope and calibration-in-the-large [16]. Discrimination was assessed by the c-statistic (equivalent to the area under the receiver operating curve) [23, 28]. The confidence intervals for both c-statistic and calibration slope and intercept were calculated through bootstrapping (i.e. iterative sampling with replacement). Additionally, to facilitate a comparison of SENSS and NETS model performance to the Neonatal Mortality Rate (NMR)-2000 [12] score findings, we also report the Brier score which reflects the combined model discrimination and calibration. These metrics are briefly described in Table 2 and explained in detail elsewhere [31].

**Table 2:** Measures for model’s performance assessment (definitions adapted from Riley et al. [31] )

1. Calibration
This is how close the predicted mortality event is close to the observed mortality event. This measure has two key components:
(a) Calibration slope
The calibration slope measures the agreement between the observed and predicted risks of the event (outcome) across the whole range of predicted values. For a perfectly calibrated model, we expect to see that, in 100 individuals with a predicted risk of *r*% from our model, *r* of the 100 truly have the outcome of interest (i.e. death in this case). The slope should ideally be 1. A slope < 1 indicates that some predictions are too extreme (e.g. predictions close to 1 are too high, and predictions close to 0 are too low), and a slope > 1 indicates predictions are too narrow. A calibration slope < 1 is often observed in validation studies, consistent with over-fitting in the original model development
(b) Calibration-in-the-large (calibration intercept)
The calibration intercept compares the mean of all predicted risks with the mean observed risk, i.e. on average how close is predicted to observed in the whole dataset. This parameter hence indicates the extent that predictions are systematically too low or too high. It can be well assessed graphically, in a plot with predictions on the *x*-axis and the observed endpoint on the *y*-axis. The observed values on the *y*-axis are 0 or 1 (e.g. dead/alive), while the predictions on the *x*-axis range between 0 and 100% with the intercept representing calibration-in-the-large
2. Discrimination
The is a measure of a prediction model’s separation between those with or without the outcome, usually represented by the c-statistic which is also known as the concordance index or, for binary outcomes, the area under the receiver operating characteristic (AUROC) curve. It gives the probability that for any randomly selected pair of individuals, one with and one without the disease (outcome), the model assigns a higher probability to the individual with the disease (outcome). A value of 1 indicates the model has perfect discrimination, while a value of 0.5 indicates the model discriminates no better than chance
3. Brier score
The Brier score captures both discrimination and calibration simultaneously, with smaller values indicating better model performance. Consider a set of events with binary outcomes (e.g. ‘death will or will not happen’). If an event comes to pass (‘death did happen’), it is assigned a value of 1 otherwise it is assigned a value of 0. Given probabilistic predictions for those events (‘.77 probability of death’), the Brier score is the mean of squared differences between those predictions and their corresponding event scores (1 s and 0 s) on the probability scale lying between 0 and 1. Larger differences between expected and observed event outcomes reflect more error in predictions, so a lower Brier score indicates greater accuracy

For objective 2 (i.e. model updating), given that simple recalibration did not resolve poor model performance (Additional file 2 [21, 32]), we refit the SENSS and NETS models and re-estimated the coefficients while applying regularisation (a technique for reducing model overfitting) using data from the 16th hospital (i.e. the models’ derivation study site). Model overfitting is when the model fits too closely to the training dataset making it unable to generalise well to new datasets. We used elastic net regularisation which combines L1 regularisation (introduces sparsity by shrinking the less important covariates’ coefficients towards zero) and L2 regularisation (minimises biassed estimates due to highly correlated independent variables) [33]. Also, to minimise model overfitting from the selection of elastic-net tunning parameters, we applied tenfold internal cross-validation repeated 20 times [34]. Cross-validation is a re-sampling procedure where the model development dataset is randomly split into an equally sized number of partitions (i.e. folds) and one of the random partitions is left out during model fitting for use as the internal validation dataset, with the model then built on the remaining portion of development dataset, and predictive performance evaluated on the left-out partition. This process is repeated with each iteration using a different partition as the validation data source. It could also include optional extra iterations to repeat the random splitting of the development dataset which would generate different folds [34]. The goal of cross-validation is assessing how accurately a predictive model might perform in practice given, for example, the different elastic net thresholds used during model fitting (i.e. thereby aiding the selection of the most optimum model hyperparameters such as regularisation parameters) [34].

The SENSS and the NETS models were fit on data collected between August 2016 and December 2020 collected from the 16th hospital. The updated SENSS and NETS model performance was evaluated on data from the other 15 hospitals (Additional file 1: Table S6). All cases included in the NETS model are a subset of those included in the SENSS model but with a treatment sheet present. Given the models are developed independently of each other, there is no substantive implication on the interpretation of findings. We provide explanations of the meaning and significance of the different datasets in Additional file 1: Table S1.

To examine the heterogeneity in model performance, we compared the updated models’ internal–external cross-validation performance where we omitted one hospital at a time using it as the validation dataset, built the model on the remaining hospitals, and evaluated the model’s discrimination and calibration performance on the hospital left out. We repeated this process with each iteration using a different hospital as the validation data source [35].

## Results

There were no noticeable differences in the level of missingness in the auxiliary variables between the derivation and temporal datasets used in the derivation study (Table 3) [13]. For the NETS predictors, the external validation dataset had a higher proportion of patients with intravenous fluids prescribed and birthweight < 1500 g (Additional file 1: Table S6). Compared to the derivation and the model updating dataset, neonates in the SENSS external dataset had a higher proportion of all predictors present except for central cyanosis and severe indrawing (Additional file 1: Table S4). There is a noticeable difference in mortality outcomes between the derivation and external validation datasets (Table 3). Around 40% of the neonates in the external validation set did not have normal birth weight, and the mortality rate was around 13–14% and around 11% of the neonates were born outside the hospital (Table 3).

**Table 3:** Characteristics of patients included in model derivation and external validation

Indicator	Levels	Derivation		Temporal validation^a^		Model updating (recalibration)^b^		External validation	
		**SENSS**^**c**^**, *n* = 5427**	**NETS**^**d**^**, *n* = 4840**	**SENSS**^**c**^**, *n* = 1627**	**NETS**^**d**^**, *n* = 1443**	**SENSS**^**c**^**, *n* = 8848**	**NETS**^**d**^**, *n* = 6610**	**SENSS**^**c**^**, *n* = 53,909**	**NETS**^**d**^**, *n* = 45,090**
		***n* (%)**	***n* (%)**	***n* (%)**	***n* (%)**	***n* (%)**	***n* (%)**	***n* (%)**	***n* (%)**
Male	Yes	2937 (54.12)	2605 (53.82)	961 (59.07)	850 (58.91)	4963 (56.09)	3713 (56.17)	29,384 (54.51)	24,719 (54.82)
	Missing	13 (0.24)	12 (0.25)	2 (0.12)	2 (0.14)	27 (0.31)	25 (0.38)	642 (1.19)	468 (1.04)
Weight	1000 g and below (ELBW)	32 (0.59)	31 (0.64)	10 (0.61)	10 (0.69)	104 (1.18)	96 (1.45)	1243 (2.31)	1083 (2.4)
	1001–1499 g (VLBW)	136 (2.51)	115 (2.38)	45 (2.77)	40 (2.77)	379 (4.28)	366 (5.54)	3844 (7.13)	3494 (7.75)
	1500–2499 g (LBW)	1180 (21.74)	1043 (21.55)	361 (22.19)	316 (21.9)	2123 (23.99)	1717 (25.98)	13,207 (24.5)	11,320 (25.11)
	2500–4000 g (NBW)	3841 (70.78)	3438 (71.03)	1125 (69.15)	1002 (69.44)	5834 (65.94)	4162 (62.97)	31,285 (58.03)	25,932 (57.51)
	> 4000 g (macrosomia)	229 (4.22)	204 (4.21)	85 (5.22)	74 (5.13)	368 (4.16)	243 (3.68)	3352 (6.22)	2478 (5.5)
	Missing	9 (0.17)	9 (0.19)	1 (0.06)	1 (0.07)	40 (0.45)	26 (0.39)	978 (1.81)	783 (1.74)
Mode of delivery	Breech	43 (0.79)	40 (0.83)	23 (1.41)	19 (1.32)	197 (2.23)	165 (2.5)	1226 (2.27)	1028 (2.28)
	Caesarean section (C/S)	2212 (40.76)	1957 (40.43)	574 (35.28)	509 (35.27)	3211 (36.29)	2251 (34.05)	18,634 (34.57)	15,540 (34.46)
	Spontaneous vaginal (SVD)^e^	3014 (55.54)	2698 (55.74)	1012 (62.2)	897 (62.16)	5157 (58.28)	4006 (60.61)	33,203 (61.59)	27,824 (61.71)
	Missing	158 (2.91)	145 (3)	18 (1.11)	18 (1.25)	283 (3.2)	188 (2.84)	846 (1.57)	698 (1.55)
Outborn^f^	Yes	123 (2.27)	107 (2.21)	60 (3.69)	57 (3.95)	495 (5.59)	439 (6.64)	6017 (11.16)	5155 (11.43)
	Missing	0 (0)	0 (0)	0 (0)	0 (0)	0 (0)	0 (0)	0 (0)	0 (0)
Apgar score (5 min)	0–3	116 (2.14)	112 (2.31)	33 (2.03)	33 (2.29)	293 (3.31)	274 (4.15)	1146 (2.13)	1005 (2.23)
	4–6	602 (11.09)	593 (12.25)	200 (12.29)	199 (13.79)	1334 (15.08)	1184 (17.91)	9121 (16.92)	7986 (17.71)
	7–10	3992 (73.56)	3918 (80.95)	1149 (70.62)	1142 (79.14)	6484 (73.28)	4868 (73.65)	38,391 (71.21)	31,531 (69.93)
	Missing	717 (13.21)	217 (4.48)	245 (15.06)	69 (4.78)	737 (8.33)	284 (4.3)	5251 (9.74)	4568 (10.13)
HIV exposure	Exposed	319 (5.88)	287 (5.93)	84 (5.16)	74 (5.13)	473 (5.35)	439 (6.64)	2145 (3.98)	1957 (4.34)
	Missing	305 (5.62)	277 (5.72)	94 (5.78)	80 (5.54)	276 (3.12)	219 (3.31)	4742 (8.8)	3826 (8.49)
Outcome	Alive^g^	4900 (90.29)	4374 (90.37)	1469 (90.29)	1299 (90.02)	8134 (91.93)	5950 (90.02)	46,358 (85.99)	38,576 (85.55)
	Dead	508 (9.36)	447 (9.24)	152 (9.34)	138 (9.56)	696 (7.87)	649 (9.82)	7486 (13.89)	6482 (14.38)
	Missing	19 (0.35)	19 (0.39)	6 (0.37)	6 (0.42)	18 (0.2)	11 (0.17)	65 (0.12)	32 (0.07)

^a^The same hospital was used at the model derivation and temporal validation stage, with the temporal validation stage using data from a specific future period

^b^Data is only from the same hospital used at derivation and temporal validation stage. Data collected between January 2016 and December 2020

^c^Data presented are before multiple imputation. The multiple imputation filled in the missing values while preserving the pattern of distribution observed in the original datasets

^d^All cases included in NETS model are subset of those included in SENSS model but with a treatment sheet present; given the models are developed independently of each other, there is no substantive implication on interpretation of findings

^e^Includes assisted vaginal deliveries (e.g. forceps, vacuum)

^f^Outborn refers to neonates admitted to the unit having been born either in another facility, at home or on the way to hospital

^g^Patients referred out of hospital recoded were also treated as being ‘alive’ at discharge

### Objective 1: SENSS and NETS model performance on the external validation dataset

**Fig. 2:**
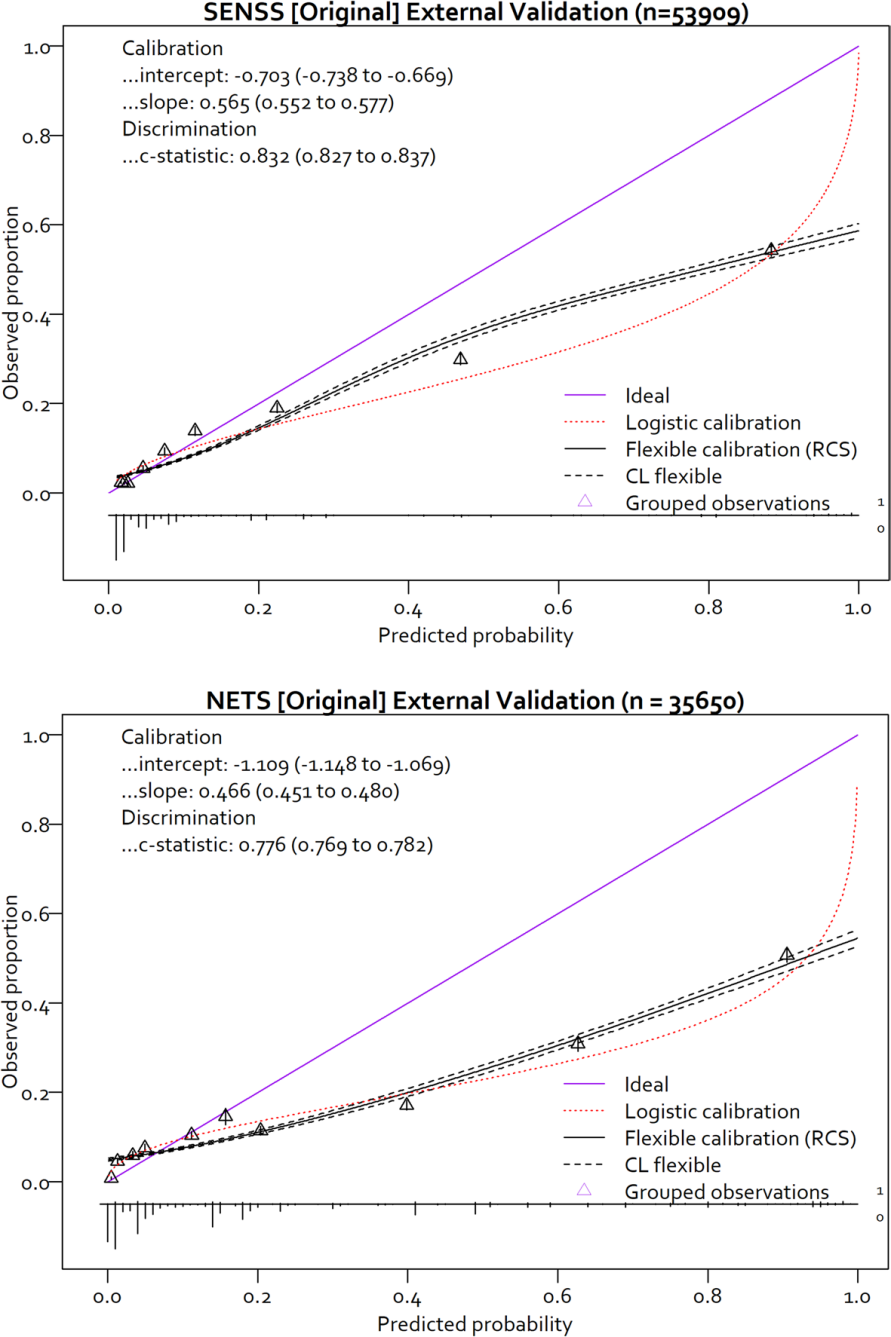
Calibration curves for the SENSS and NETS model in the external validation dataset. SENSS, Score for Essential Neonatal Symptoms and Signs; NETS, Neonatal Essential Treatment Score; RCS, restricted cubic splines; CL, confidence limits (95%). Calibration curves generated using the CalibrationCurves package in R [36]

The c-statistic (discrimination) for SENSS model was 0.832 (95% CI 0.827 to 0.837) and 0.776 (95% CI 0.769 to 0.782) for the NETS model. The calibration of the original SENSS and NETS models (Table 1) was poor as reflected by the calibration intercept and slope (Fig. 2). The estimated mortality risks from both models were too extreme (slope < 1) and tended towards overestimation (intercept < 0), especially as the observed outcome event rate increased.

### Objective 2: SENSS and NETS model updating

**Fig. 3:**
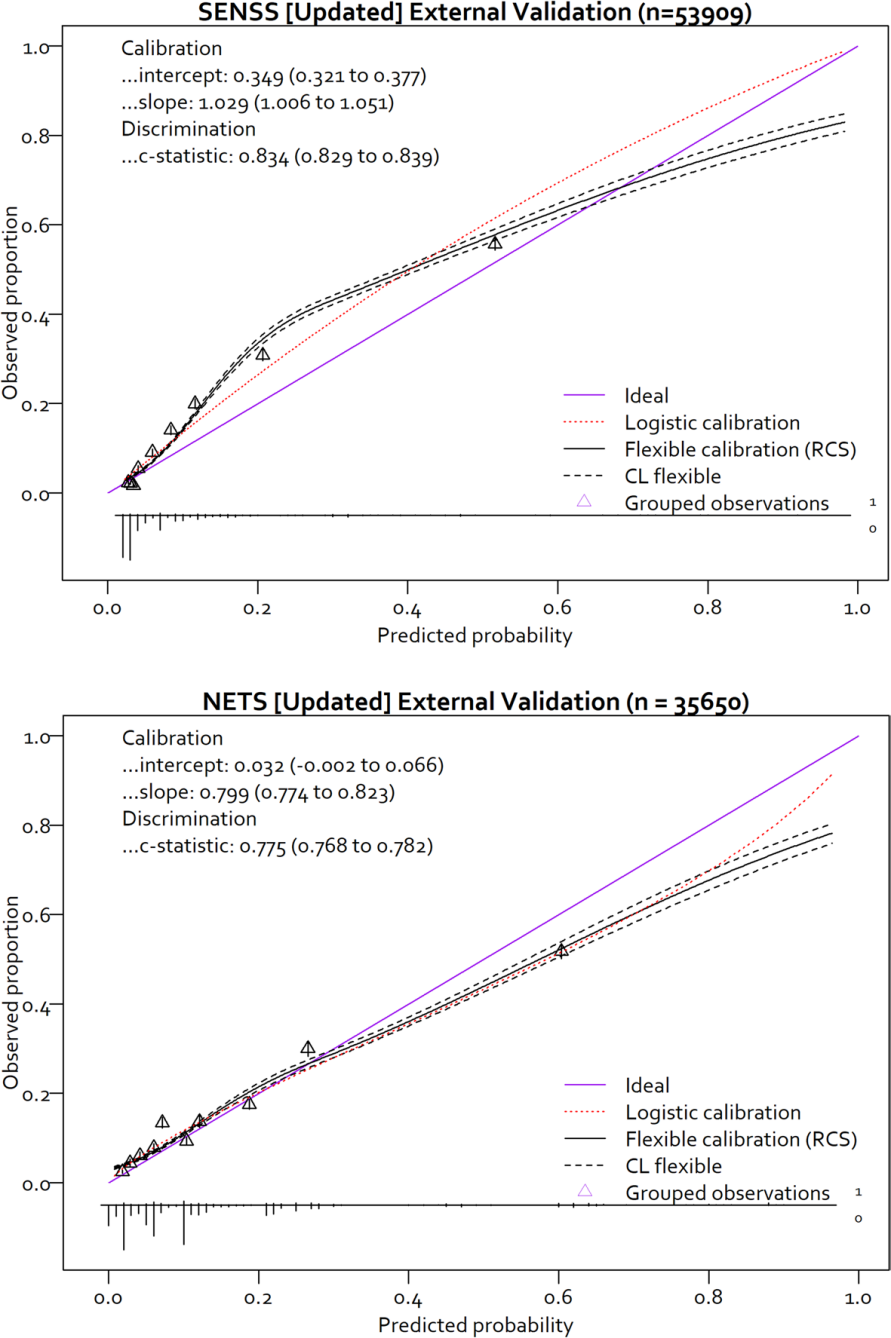
Calibration curves for the updated SENSS and NETS model in the external validation dataset. SENSS, Score for Essential Neonatal Symptoms and Signs; NETS, Neonatal Essential Treatment Score; RCS, restricted cubic splines; CL, confidence limits (95%). Calibration curves generated using the CalibrationCurves package in R [36]

After model updating, SENSS and NETS model calibration intercepts improved to 0.349 (95% CI 0.321 to 0.377) and 0.032 (95% CI − 0.002 to 0.066), respectively. The updated SENSS unlike the updated NETS model was still suggestive of mortality risk underestimation (Fig. 3). The calibration slopes also improved to 1.029 (95% CI 1.006 to 1.051) for SENSS and 0.799 (95% CI 0.744 to 0.823) for NETS models (Fig. 3). The c-statistic from both models after did not show any statistically significant improvement. The Brier score of the SENSS and NETS models was 0.093 (95% CI 0.084 to 0.104) and 0.105 (95% CI 0.095 to 0.116), respectively. The coefficients from the updated NETS and SENSS models are illustrated in Table 4.

**Table 4:** Logistic regression models for NETS and SENSS after model updating

SENSS:
Linear predictor (*LP*_SENSS_) = − 3.4635 + 2.8734 * ELBW + 1.7696 * VLBW + 0.3352 * LBW − 0.2396 * macrosomia − 0.0685 * male + 0.6400 * difficulty feeding + 0.5300 * convulsions + 1.5078 * indrawing + 1.0633 * cyanosis + 0.9583 * floppy unable to suck
NETS:
Linear predicator (*LP*_NETS_) = − 3.5246 + 4.2077 * ELBW + 2.6112 * VLBW + 0.8759 * LBW + 0.000 * macrosomia − 0.0667 * male + 0.5962 * antibiotics + 0.8095 * fluids − 1.2843 * feeds + 0.1522 * oxygen + 1.1303 * phenobarbital

For each variable, the presence of the indicator takes a value of 1, and the absence takes a value of 0. The coefficients are summated to give the linear predictor, which is then converted to the predicted probability of in-hospital mortality

*ELBW* Extremely low birth weight, *LBW* Low birth weight, *LP* Linear predictor, *NETS* Neonatal Essential Treatment Score, *SENSS* Score of Essential Neonatal Symptoms and Signs, *VLBW* Very low birth weight

**Fig. 4:**
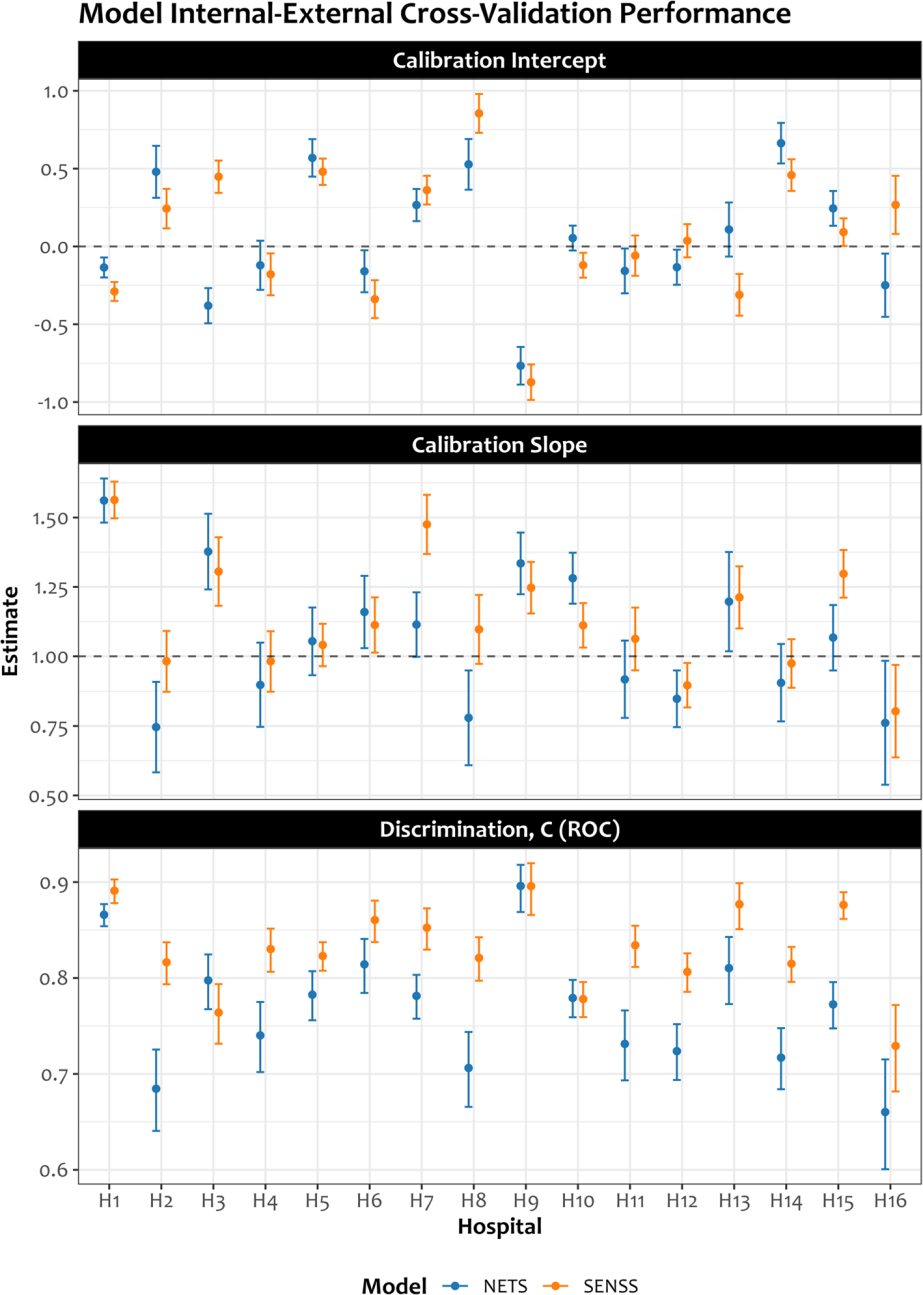
Heterogeneity in model performance from internal–external cross-validation (IECV) approach. SENSS, Score for Essential Neonatal Symptoms and Signs; NETS, Neonatal Essential Treatment Score

Figure 4 illustrates the findings from the sensitivity analysis applying internal–external cross-validation (IECV) to explore the heterogeneity in model performance and see in which hospitals the models ‘work’ and ‘do not work’ based on the performance measures in Table 2. From the IECV findings (Fig. 4), 7/16 and 6/16 hospitals had a calibration intercept suggestive of underestimation of the estimated mortality risk when using SENSS and NETS models, respectively; 6/16 hospitals had a calibration intercept suggestive of overestimation of the estimated mortality risk when using SENSS and NETS models.

Four out of 16 hospitals had a calibration slope indicating that the estimated NETS mortality risks were too extreme, i.e. too high for patients who are at high risk and too low for patients who are at low risk; 6/16 and 8/16 hospitals had a slope > 1 for NETS and SENSS models, respectively, suggesting that the mortality risk estimates from these models for these hospitals are too moderate. The c-statistic of only 9/16 hospitals for the SENSS model and 2/16 for the NETS model had a 95% confidence interval that was above 0.8 (Fig. 4).

## Discussion

The NETS and SENSS models demonstrated improved performance after updating the initial specified models [13]. At initial external validation, calibration-in-the-large (calibration intercept) for NETS was − 1.109 (95% CI − 1.148 to − 1.069) and that for SENSS was − 0.703 (95% CI − 0.738 to − 0.669). The calibration slope for NETS was 0.466 (95% CI 0.451 to 0.480), and for SENSS, it was 0.565 (95% CI 0.552 to 0.577). After updating the data used at the derivation stage while applying cross-validation, model regularisation, and imputation fitting procedures, SENSS and NETS model calibration intercepts improved to 0.349 (95% CI 0.321 to 0.377) and 0.032 (95% CI − 0.002 to 0.066), respectively, and their calibration slopes also improved to 1.029 (95% CI 1.006 to 1.051) for SENSS and 0.799 (95% CI 0.774 to 0.823) for NETS model.

Our analyses sought to address the previous shortcomings of external validation studies by (i) reporting model calibration using various performance statistics (Brier score, calibration slope, calibration intercept), (ii) explicitly highlighting the treatment of missing data, (iii) making clear the version(s) of the original model being evaluated, and (iv) using a big sample size for analysis [31]. The pre-selection of model predictors was based on their availability in typical LMICs clinical practice [10, 37] and represents the ideal case of using a limited number of predictors in the final models [23]. As more parsimonious models (fewer predictors), the NETS and SENSS might have better predictive performance while being reflective of many LMIC neonatal contexts [23]. Apart from the consensus of 0.8 as a threshold marker of good discrimination, there is no agreement on cut-off thresholds for good calibration. We have therefore adopted calibration plots and report the calibration slopes and intercepts [16]. However, we also report alternative scores (i.e. Brier score [38]) to aid comparison to other studies’ findings [12].

Model updating would help where the model has poor calibration but good discrimination in the external validation dataset, where the difference between the development and validation datasets is in both the predictor and observed outcome frequencies and where the strength of the association between predictors and the outcome might be substantially different in the new population [21]; this was the case for the SENSS and NETS models in this study, with calibration slopes suggesting that the estimated mortality risks are too extreme (Fig. 2, Additional file 2: Fig. S1). A possible reason for the difference in performance might be due to the strength of the association between some predictors and the outcome might be substantially different in the new population [21]. For example, the proportion of deaths per predictor for both models in the external validation dataset varied substantively compared to the derivation dataset (Additional file 1: Tables S7 and S8) [13]. This calibration performance difference might also be due to dissimilar case fatality—exacerbated by the difference in the level of predictor missingness—between the original derivation and the new external validation datasets.

Our published review showed that there are arguably no ‘gold standard’ clinical prediction models for neonatal mortality in LMIC. Existing models either (1) are often developed in intensive care units, (2) lack external validation, (3) suffer from widespread methodological limitations, (4) have low certainty in the quality of evidence on their performance, or (5) are developed to predict risk at the population level as opposed to the in-hospital [8, 12, 39]. These neonatal prediction models (including commonly used models from high-income settings) tend to rely on predictors that are laboratory- and therapy-derived that are often unavailable in care settings in LMICs; they are unlikely to be externally validated in settings such as Kenya [8, 40, 41]. Comparison of the SENSS and NETS external discrimination and calibration performance to the performance of these previously identified models’ is thus ill-advised since these measures are generated from different patient cohorts [8].

The most similar (and recently validated) score to NETS and SENSS is the NMR-2000, whose external validation sample size from LMICs was 457 patients from The Gambia and relied on the presence of pulse oximetry [12]. When diagnostic tools (e.g. pulse oximetry) become unavailable in routine practice, which is often the case in typical LMIC settings in SSA, prediction models like NMR-2000 are rendered unusable [12].

The most valuable characteristic of a prediction model performance is its generalisability to an external population, where calibration-in-the-large is highly relevant. From Fig. 2, the original SENSS and NETS models’ calibration on the external dataset were noticeably poor and would have tended towards overestimating mortality risk despite being ‘simple’ models [16]. Even though the magnitude of the proportion of deaths across the external validation dataset for both NETS and SENSS datasets was similar, the differences in the proportion of deaths for both models were higher in the external validation dataset compared to the derivation dataset (Additional file 1: Tables S3 and S4), which might have contributed to the differences in model calibration observed at external validation. This might have resulted in a case-mix difference due to random variation before model updating was done.

Our approach of applying penalised regression techniques (i.e. regularisation) and repeated internal cross-validation was able to reduce model overfitting and improve calibration as evidenced by the better calibration intercepts and slopes (Fig. 3). From the IECV findings evaluating heterogeneity in model performance (Fig. 4), hospital-specific differences in the recognition of signs and symptoms of severe illness (for SENSS), coupled with differences in case-mix influencing treatment outcomes, might have been key factors contributing to the differences observed in model performance. The heterogeneity of the NETS model discrimination is likely from systematic differences in hospital practices contributed to by changes in junior clinician rotations. IECV performance was relatively better for the SENSS model, likely because recognition of signs and symptoms of severe illness, which requires different arguably less complex clinical skills than prescription of treatments (NETS predictors), might not vary so much across hospitals.

A key strength of this study is our use of a large and purposely selected neonatal dataset from routine clinical settings from geographically dispersed hospitals for external model validation (Fig. 1). Such datasets, with associated external validation studies, are quite uncommon in LMIC settings [18, 31]. We also transitioned from temporal validation of the NETS and SENSS models [13] to geographical external validation to enhance generalisability in more neonatal units and better address heterogeneity in model performance across populations [31]. A key limitation of this study is that variation in treatments may be influenced by time, resources available, and level of care provided [8]. Nonetheless, the use of standard clinical guidelines [10] helps to reduce such variation, although routine model updating needs to also be considered as clinical practice changes over time [16]. Also, while purposively sampling in partnership with the Kenyan MoH generated the CIN hospitals which have varied NBU characteristics, CIN hospitals perhaps have higher-quality clinical data from NBUs than is available from all NBUs in Kenya.

## Conclusion

The SENSS and the NETS are simplified prediction models relying on basic routine clinical data recorded by duty clinicians, validated for low-resource settings, to accurately predict in-hospital mortality among neonates with varying birth weights. By enabling healthcare workers in LMIC settings to quickly assess mortality risk (e.g. using a neonate’s signs, symptoms, and treatments to calculate mortality probability), these scores could help improve early recognition of illness severity and rapid initiation of evidence-based interventions, crucial for the neonates’ survival. However, more research is needed on how best to translate SENSS and NETS models to (a) simplified clinical prediction or decision rules for clinical use and (b) test them using intervention studies to determine their impact on patient outcomes and cost-effectiveness [14]. They are however well-suited for aiding decision-makers with case-mix adjustment decisions.

## Supplementary Information

**Additional file 1: Table S1.** Different types and meanings of datasets in predictive modelling. **Table S2.** Essential treatments prescribed at admission that were included as predictors. **Table S3.** Symptoms and signs of severe illness considered as candidate predictors. **Table S4.** Distribution of predictors for patients included in the SENSS model derivation, updating and external validation. **Table S5.** Distribution of predictors for patients included in the NETS model derivation, updating and external validation. **Table S6.** Hospital Specific Candidate Predictor Summaries. **Table S7.** Distribution of predictors and missingness in SENSS external validation dataset by in-hospital mortality. **Table S8.** Distribution of predictors in NETS external validation dataset by in-hospital mortality.

**Additional file 2.** Model recalibration approaches evaluated.

## Data Availability

Data are available upon reasonable request. The source data are owned by the Kenyan Ministry of Health, County Governments, and as the data might be used to de-identify hospitals, the study authors are not permitted to share the source data directly. Users who wish to reuse the source data can make a request initially through the KEMRI-Wellcome Trust Research Programme data governance committee. This committee will supply the contact information for the KEMRI Scientific and Ethical Review Unit, County Governments, and individual hospitals as appropriate. The KEMRI-Wellcome Trust Research data governance committee can be contacted at dgc@kemri-wellcome.org.

## Abbreviations

CI: Confidence interval
ELBW: Extremely low birth weight
HRIO: Hospital Records Information Officer
IECV: Internal-external cross-validation
KEMRI: Kenya Medical Research Institute
LBW: Low birth weight
LMICs: Low- and middle-income countries
LP: Linear predictor
MAR: Missing at random
MICE: Multivariate imputation by chained equations
MoH: Ministry of Health
NETS: Neonatal Essential Treatment Score
NMR: Neonatal mortality rate
RCS: Restricted cubic splines
SENSS: Score of Essential Neonatal Symptoms and Signs
SERU: KEMRI’s Scientific and Ethics Review Unit
SSA: Sub-Saharan Africa
VLBW: Very low birth weight
WHO: World Health Organization

## References

[1] Hug L (2019). National, regional, and global levels and trends in neonatal mortality between 1990 and 2017, with scenario-based projections to 2030: a systematic analysis. Lancet Glob Health.

[2] United Nations General Assembly (2016). Transforming our world: the 2030 agenda for sustainable development.

[3] Murphy GA (2018). Effective coverage of essential inpatient care for small and sick newborns in a high mortality urban setting: a cross-sectional study in Nairobi City County. Kenya BMC Med.

[4] Kihuba E (2014). Assessing the ability of health information systems in hospitals to support evidence-informed decisions in Kenya. Glob Health Action.

[5] Hagel C (2020). Data for tracking SDGs: challenges in capturing neonatal data from hospitals in Kenya. BMJ Glob Health.

[6] Jencks SF, Dobson A (1987). Refining case-mix adjustment. N Engl J Med.

[7] Steyerberg EW (2013). Prognosis Research Strategy (PROGRESS) 3: prognostic model research. PLoS Med.

[8] Aluvaala J (2017). A systematic review of neonatal treatment intensity scores and their potential application in low-resource setting hospitals for predicting mortality, morbidity and estimating resource use. Syst Rev.

[9] Moons KG (2015). Transparent Reporting of a multivariable prediction model for Individual Prognosis or Diagnosis (TRIPOD): explanation and elaboration. Ann Intern Med.

[10] Ministry of Health (2016). Basic paediatric protocols.

[11] Tuti T (2016). Innovating to enhance clinical data management using non-commercial and open source solutions across a multi-center network supporting inpatient pediatric care and research in Kenya. J Am Med Inform Assoc.

[12] Medvedev MM (2020). Development and validation of a simplified score to predict neonatal mortality risk among neonates weighing 2000 g or less (NMR-2000): an analysis using data from the UK and The Gambia. Lancet Child Adolesc Health.

[13] Aluvaala J (2020). Prediction modelling of inpatient neonatal mortality in high-mortality settings. Arch Dis Child.

[14] Kent P (2020). A conceptual framework for prognostic research. BMC Med Res Methodol.

[15] Altman DG, Royston P (2000). What do we mean by validating a prognostic model?. Stat Med.

[16] Van Calster B (2019). Calibration: the Achilles heel of predictive analytics. BMC Med.

[17] Collins GS (2015). T*ransparent* Reporting of a multivariable prediction model for Individual Prognosis Or Diagnosis (TRIPOD*)*: the TRIPOD statement. Circulation.

[18] Maina M (2018). Using a common data platform to facilitate audit and feedback on the quality of hospital care provided to sick newborns in Kenya. BMJ Glob Health.

[19] Irimu G (2021). Neonatal mortality in Kenyan hospitals: a multisite, retrospective, cohort study. BMJ Glob Health.

[20] Harris PA (2009). Research electronic data capture (REDCap)—a metadata-driven methodology and workflow process for providing translational research informatics support. J Biomed Inform.

[21] Su T-L (2018). A review of statistical updating methods for clinical prediction models. Stat Methods Med Res.

[22] Aluvaala J (2015). Assessment of neonatal care in clinical training facilities in Kenya. Arch Dis Child.

[23] Vergouwe Y, Steyerberg EW, Eijkemans MJ, Habbema JDF. Substantial effective sample sizes were required for external validation studies of predictive logistic regression models. J Clin Epidemiol. 2005:58(5):475–83. 10.1016/j.jclinepi.2004.06.017 15845334

[24] Riley RD (2019). Minimum sample size for developing a multivariable prediction model: part II-binary and time-to-event outcomes. Stat Med.

[25] Ogundimu EO, Altman DG, Collins GS (2016). Adequate sample size for developing prediction models is not simply related to events per variable. J Clin Epidemiol.

[26] Harrell FE, Dupont C. Hmisc: harrell miscellaneous. R package version. 2008:3(2):437.

[27] Hardt J, Herke M, Leonhart R (2012). Auxiliary variables in multiple imputation in regression with missing X: a warning against including too many in small sample research. BMC Med Res Methodol.

[28] Steyerberg EW, Vergouwe Y (2014). Towards better clinical prediction models: seven steps for development and an ABCD for validation. Eur Heart J.

[29] White IR, Royston P, Wood AM (2011). Multiple imputation using chained equations: issues and guidance for practice. Stat Med.

[30] Honaker J, King G, Blackwell M (2011). Amelia II: a program for missing data. J Stat Softw.

[31] Riley RD (2016). External validation of clinical prediction models using big datasets from e-health records or IPD meta-analysis: opportunities and challenges. BMJ.

[32] Van Calster B (2017). Validation and updating of risk models based on multinomial logistic regression. Diagn Progn Res.

[33] Zou H, Hastie T (2005). Regularization and variable selection via the elastic net. J R Stat Soc Series B Stat Methodol.

[34] Kohavi Ron. "A study of cross-validation and bootstrap for accuracy estimation and model selection." In Ijcai. 1995;14(2):1137–45.

[35] Takada T (2021). Internal-external cross-validation helped to evaluate the generalizability of prediction models in large clustered datasets. J Clin Epidemiol.

[36] Bavo De Cock. CalibrationCurves. Plots calibration curves and computes statistics for assessing calibration performance 2019 [cited 2022 20th February ]; Available from: https://github.com/BavoDC/CalibrationCurves.

[37] Opiyo N, English M (2011). What clinical signs best identify severe illness in young infants aged 0–59 days in developing countries? A systematic review. Arch Dis Child.

[38] Rufibach K (2010). Use of Brier score to assess binary predictions. J Clin Epidemiol.

[39] Houweling TA (2019). A prediction model for neonatal mortality in low-and middle-income countries: an analysis of data from population surveillance sites in India, Nepal and Bangladesh. Int J Epidemiol.

[40] Dorling J, Field D, Manktelow B (2005). Neonatal disease severity scoring systems. Arch Dis Child Fetal Neonatal Ed.

[41] Aluvaala J, Collins GS, Maina B, et al. Competing risk survival analysis of time to in-hospital death or discharge in a large urban neonatal unit in Kenya. Wellcome Open Res. 2019;4:96. 10.12688/wellcomeopenres.15302.1 PMC6611136 31289756

