# Supplementary material for: External validation of inpatient neonatal mortality prediction models in high-mortality settings
Source: BMC Med. 2022 Aug 3;20:236. doi: 10.1186/s12916-022-02439-5 (PMC9347100; doi:10.1186/s12916-022-02439-5)
Supplement: Supplementary file 1 — Additional file 1: Table S1. Different types and meanings of datasets in predictive modelling. Table S2. Essential treatments prescribed at admission that were included as predictors. Table S3. Symptoms and signs of severe illness considered as candidate predictors. Table S4. Distribution of predictors for patients included in the SENSS model derivation, updating and external validation. Table S5. Distribution of predictors for patients included in the NETS model derivation, updating and external validation. Table S6. Hospital Specific Candidate Predictor Summaries. Table S7. Distribution of predictors and missingness in SENSS external validation dataset by in-hospital mortality. Table S8. Distribution of predictors in NETS external validation dataset by in-hospital mortality. [file 12916_2022_2439_MOESM1_ESM.docx]

## **Additional file 1**

| Table S1: Different types and meanings of datasets in predictive modelling | | |
| --- | --- | --- |
| Dataset name | **Dataset Description** | **Dataset utility** |
| Development dataset | The dataset that is used to fit the statistical model. | Used to derive model covariate beta coefficients (i.e., model weights) |
| Internal validation dataset | The dataset that is used for model hyperparameter tunning which is a sub-sample of the model development dataset. (i.e., patients are from the same underlying population as the development dataset) | Useful for tuning and finding optimum model hyper-parameters (i.e., parameters external to the development dataset that control the statistical model learning process) |
| Temporal validation dataset | Dataset from the same setting (e.g., hospitals/ organisation units) as those used in the development dataset where patients are sampled at a later (or earlier) time point. | Useful for assessing a model’s transportability* over time |
| External validation dataset | Dataset of patients from the different settings (e.g., hospitals/organisation units, geographical regions, or countries) to those used in the development dataset. | Useful for assessing a model’s geographic reproducibility and generalisability |
| Model updating dataset | Dataset used to fine-tunning the existing clinical prediction models already developed. | Used to update model covariate beta coefficients (i.e., model weights) to improve model transportability* |
| Note:  *Transportability term refers to a model that maintains its performance in a population that is different from that in which it was developed | | |

| Table S2: Essential treatments prescribed at admission that were included as predictors | | | |
| --- | --- | --- | --- |
| Variable name | **Description** | **Definition** | |
|  |  | **Yes** | **No*** |
| Intravenous antibiotics | Penicillin AND gentamicin are first line antibiotics prescribed together for possible severe bacterial infection. | Prescribed | Not prescribed |
| Intravenous fluids | Any prescription of an infusion of  intravenous fluid e.g., 10% Dextrose | Prescribed | Not prescribed |
| Enteral feeds | Expressed breast milk or formula fed  through nasogastric tube or cup | Prescribed | Not prescribed |
| Oxygen | Supplemental oxygen provided by any method e.g., nasal cannula | Prescribed | Not prescribed |
| Phenobarbital | Prescription of phenobarbital | Prescribed | Not prescribed |
| *Reference category in the model | | | |

| Table S3: Symptoms and signs of severe illness considered as candidate predictors | | | |
| --- | --- | --- | --- |
| Variable name | **Description^ǂ^** | **Definition** | |
|  |  | **Yes** | **No*** |
| Difficulty feeding | Symptom: reported history of difficulty feeding | Present | Absent |
| Convulsions | Symptom: reported episode(s) of convulsions | Present | Absent |
| Central cyanosis | Sign: bluish or greyish discolouration of the tongue | Present | Absent |
| Severe Indrawing | Sign: severe (deep) lower chest indrawing | Present | Absent |
| Floppy/inability to suck | Sign: measure of altered consciousness in young infants (age 0-59 days) | Present | Absent |
| *Reference category in the model. Documented that the patient did not have the symptom or sign present  ^ǂ^Adapted from Opiyo N, English M. What clinical signs best identify severe illness in young infants aged 0-59 days in developing countries? A systematic review. Archives of Disease in Childhood. 2011;96(11):1052-9. | | | |

| Table S4: Distribution of predictors for patients included in the SENSS model derivation, updating and external validation | | | | | |
| --- | --- | --- | --- | --- | --- |
|  | | **Derivation**  **(N = 5427)** | **Temporal Validation**  **(N = 1627)** | **Model Updating***  **(N = 8848)** | **External Validation**  **(N = 53909)** |
| Predictor | **Levels** | **n (%)** | **n (%)** | **n (%)** | **n (%)** |
| Male | Yes | 2937 (54.12) | 961 (59.07) | 4963 (56.09) | 29384 (54.51) |
|  | Missing | 13 (0.24) | 2 (0.12) | 27 (0.31) | 642 (1.19) |
| Birth weight | 1000 grams & below (ELBW) | 32 (0.59) | 10 (0.61) | 104 (1.18) | 1243 (2.31) |
|  | 1001 - 1499 grams (VLBW) | 136 (2.51) | 45 (2.77) | 379 (4.28) | 3844 (7.13) |
|  | 1500 - 2499 grams (LBW) | 1180 (21.74) | 361 (22.19) | 2123 (23.99) | 13207 (24.5) |
|  | 2500 - 4000 grams (NBW) | 3841 (70.78) | 1125 (69.15) | 5834 (65.94) | 31285 (58.03) |
|  | > 4000 grams (Macrosomia) | 229 (4.22) | 85 (5.22) | 368 (4.16) | 3352 (6.22) |
|  | Missing | 9 (0.17) | 1 (0.06) | 40 (0.45) | 978 (1.81) |
| Difficulty feeding | Yes | 225 (4.15) | 134 (8.24) | 971 (10.97) | 7741 (14.36) |
|  | Missing | 575 (10.6) | 199 (12.23) | 1401 (15.83) | 7248 (13.44) |
| Convulsions | Yes | 29 (0.53) | 25 (1.54) | 218 (2.46) | 1975 (3.66) |
|  | Missing | 571 (10.52) | 199 (12.23) | 1385 (15.65) | 6845 (12.7) |
| Central cyanosis | Yes | 222 (4.09) | 64 (3.93) | 450 (5.09) | 1950 (3.62) |
|  | Missing | 570 (10.5) | 216 (13.28) | 1680 (18.99) | 3013 (5.59) |
| Severe indrawing | Yes | 429 (7.9) | 62 (3.81) | 314 (3.55) | 3112 (5.77) |
|  | Missing | 620 (11.42) | 234 (14.38) | 2243 (25.35) | 5299 (9.83) |
| Floppy/inability to suck | Yes | 530 (9.77) | 359 (22.07) | 2561 (28.94) | 17389 (32.26) |
|  | Missing | 852 (15.7) | 217 (13.34) | 1300 (14.69) | 7886 (14.63) |
| Outcome | Alive | 4900 (90.29) | 1469 (90.29) | 8134 (91.93) | 46358 (85.99) |
|  | Dead | 508 (9.36) | 152 (9.34) | 696 (7.87) | 7486 (13.89) |
|  | Missing | 19 (0.35) | 6 (0.37) | 18 (0.2) | 65 (0.12) |
| *Data only from the same hospital used at derivation and temporal validation stage. Data collected between January 2016 – December 2020. | | | | | |

| Table S5: Distribution of predictors for patients included in the NETS model derivation, updating and external validation | | | | | |
| --- | --- | --- | --- | --- | --- |
|  | | **Derivation**  **(N = 4840)** | **Temporal Validation**  **(N = 1443)** | **Model Updating***  **(N = 6610)** | **External Validation**  **(N = 45090)** |
| Indicator | **Levels** | **n (%)** | **n (%)** | **n (%)** | **n (%)** |
| Male | Yes | 2605 (53.82) | 850 (58.91) | 3713 (56.17) | 24719 (54.82) |
|  | Missing | 12 (0.25) | 2 (0.14) | 25 (0.38) | 468 (1.04) |
| Birth weight | 1000 grams & below (ELBW) | 31 (0.64) | 10 (0.69) | 96 (1.45) | 1083 (2.4) |
|  | 1001 - 1499 grams (VLBW) | 115 (2.38) | 40 (2.77) | 366 (5.54) | 3494 (7.75) |
|  | 1500 - 2499 grams (LBW) | 1043 (21.55) | 316 (21.9) | 1717 (25.98) | 11320 (25.11) |
|  | 2500 - 4000 grams (NBW) | 3438 (71.03) | 1002 (69.44) | 4162 (62.97) | 25932 (57.51) |
|  | > 4000 grams (Macrosomia) | 204 (4.21) | 74 (5.13) | 243 (3.68) | 2478 (5.5) |
|  | Missing | 9 (0.19) | 1 (0.07) | 26 (0.39) | 783 (1.74) |
| Enteral feeding | Yes | 1380 (28.51) | 598 (41.44) | 1676 (25.36) | 6053 (13.42) |
|  | Missing | 0 (0) | 0 (0) | 0 (0) | 0 (0) |
| Intravenous antibiotics | Yes | 1715 (35.43) | 726 (50.31) | 3914 (59.21) | 21125 (46.85) |
|  | Missing | 0 (0) | 0 (0) | 0 (0) | 0 (0) |
| Intravenous fluids | Yes | 869 (17.95) | 495 (34.3) | 2812 (42.54) | 19651 (43.58) |
|  | Missing | 0 (0) | 0 (0) | 888 (13.43) | 8645 (19.17) |
| Oxygen prescribed | Yes | 295 (6.1) | 105 (7.28) | 1462 (22.12) | 7700 (17.08) |
|  | Missing | 0 (0) | 0 (0) | 0 (0) | 0 (0) |
| Phenobarbital prescribed | Yes | 141 (2.91) | 77 (5.34) | 664 (10.05) | 4103 (9.1) |
|  | Missing | 0 (0) | 0 (0) | 0 (0) | 0 (0) |
| Outcome | Alive | 4374 (90.37) | 1299 (90.02) | 5950 (90.02) | 38576 (85.55) |
|  | Dead | 447 (9.24) | 138 (9.56) | 649 (9.82) | 6482 (14.38) |
|  | Missing | 19 (0.39) | 6 (0.42) | 11 (0.17) | 32 (0.07) |
| * Data only from the same hospital used at derivation and temporal validation stage. Data collected between January 2016 – December 2020. | | | | | |

| Table S6: Hospital Specific Candidate Predictor Summaries^ǂ^ | | | | | | | | | | | | | | | | |
| --- | --- | --- | --- | --- | --- | --- | --- | --- | --- | --- | --- | --- | --- | --- | --- | --- |
| Indicator | **H1*** | **H2** | **H3** | **H4** | **H5** | **H6** | **H7** | **H8** | **H9** | **H10** | **H11** | **H12** | **H13** | **H14** | **H15** | **H16** |
|  |  |  |  |  |  |  |  |  |  |  |  |  |  |  |  |  |
| SENSS candidate predictors | | | | | | | | | | | | | | | | |
| n | 15902 | 1911 | 4188 | 1881 | 4285 | 3462 | 5493 | 1716 | 6587 | 6101 | 2338 | 3415 | 3370 | 2748 | 5356 | 1058 |
| Mortality Rate | 1356 (8.53) | 397 (20.77) | 455 (10.86) | 364 (19.35) | 893 (20.84) | 384 (11.09) | 594 (10.81) | 421 (24.53) | 390 (5.92) | 837 (13.72) | 349 (14.93) | 543 (15.9) | 306 (9.08) | 671 (24.42) | 725 (13.54) | 157 (14.84) |
| Male | 8861 (55.72) | 1049 (54.89) | 2289 (54.66) | 1023 (54.39) | 2176 (50.78) | 1866 (53.9) | 2880 (52.43) | 1004 (58.51) | 3648 (55.38) | 3472 (56.91) | 1266 (54.15) | 1965 (57.54) | 1810 (53.71) | 1474 (53.64) | 2873 (53.64) | 589 (55.67) |
| *Birth weight* |  |  |  |  |  |  |  |  |  |  |  |  |  |  |  |  |
| 1000 grams & Below (ELBW) | 146 (0.92) | 45 (2.35) | 67 (1.6) | 84 (4.47) | 154 (3.59) | 75 (2.17) | 51 (0.93) | 41 (2.39) | 103 (1.56) | 176 (2.88) | 90 (3.85) | 106 (3.1) | 68 (2.02) | 75 (2.73) | 93 (1.74) | 15 (1.42) |
| 1001 - 1499 grams (VLBW) | 560 (3.52) | 207 (10.83) | 176 (4.2) | 221 (11.75) | 439 (10.25) | 189 (5.46) | 271 (4.93) | 177 (10.31) | 274 (4.16) | 468 (7.67) | 225 (9.62) | 383 (11.22) | 167 (4.96) | 274 (9.97) | 289 (5.4) | 84 (7.94) |
| 1500 - 2499 grams (LBW) | 3664 (23.04) | 446 (23.34) | 747 (17.84) | 587 (31.21) | 1225 (28.59) | 817 (23.6) | 1261 (22.96) | 460 (26.81) | 1275 (19.36) | 1618 (26.52) | 741 (31.69) | 936 (27.41) | 741 (21.99) | 784 (28.53) | 1231 (22.98) | 338 (31.95) |
| 2500 - 4000 grams (NBW) | 10800 (67.92) | 955 (49.97) | 3039 (72.56) | 857 (45.56) | 2019 (47.12) | 2183 (63.06) | 3548 (64.59) | 850 (49.53) | 4565 (69.3) | 3165 (51.88) | 1170 (50.04) | 1741 (50.98) | 2050 (60.83) | 1424 (51.82) | 3145 (58.72) | 574 (54.25) |
| > 4000 grams (Macrosomia) | 682 (4.29) | 66 (3.45) | 154 (3.68) | 62 (3.3) | 354 (8.26) | 149 (4.3) | 348 (6.34) | 45 (2.62) | 337 (5.12) | 646 (10.59) | 77 (3.29) | 223 (6.53) | 294 (8.72) | 115 (4.18) | 452 (8.44) | 30 (2.84) |
|  |  |  |  |  |  |  |  |  |  |  |  |  |  |  |  |  |
| Difficulty feeding | 1330 (8.36) | 551 (28.83) | 105 (2.51) | 585 (31.1) | 975 (22.75) | 513 (14.82) | 181 (3.3) | 314 (18.3) | 901 (13.68) | 971 (15.92) | 275 (11.76) | 554 (16.22) | 245 (7.27) | 709 (25.8) | 653 (12.19) | 209 (19.75) |
| Convulsions | 272 (1.71) | 163 (8.53) | 40 (0.96) | 154 (8.19) | 275 (6.42) | 141 (4.07) | 50 (0.91) | 66 (3.85) | 253 (3.84) | 97 (1.59) | 91 (3.89) | 124 (3.63) | 56 (1.66) | 221 (8.04) | 163 (3.04) | 81 (7.66) |
| Central cyanosis | 736 (4.63) | 68 (3.56) | 115 (2.75) | 114 (6.06) | 85 (1.98) | 159 (4.59) | 73 (1.33) | 92 (5.36) | 134 (2.03) | 130 (2.13) | 87 (3.72) | 149 (4.36) | 328 (9.73) | 128 (4.66) | 271 (5.06) | 17 (1.61) |
| Severe indrawing | 805 (5.06) | 140 (7.33) | 112 (2.67) | 217 (11.54) | 180 (4.2) | 289 (8.35) | 133 (2.42) | 87 (5.07) | 257 (3.9) | 475 (7.79) | 98 (4.19) | 256 (7.5) | 140 (4.15) | 355 (12.92) | 316 (5.9) | 57 (5.39) |
| Floppy/inability to suck | 3450 (21.7) | 801 (41.92) | 378 (9.03) | 949 (50.45) | 1816 (42.38) | 1451 (41.91) | 745 (13.56) | 586 (34.15) | 1888 (28.66) | 3377 (55.35) | 685 (29.3) | 1147 (33.59) | 811 (24.07) | 1072 (39.01) | 1325 (24.74) | 358 (33.84) |
|  |  |  |  |  |  |  |  |  |  |  |  |  |  |  |  |  |
| NETS candidate predictors | | | | | | | | | | | | | | | | |
| n | 11938 | 923 | 3535 | 1202 | 1994 | 2259 | 3098 | 897 | 5588 | 5916 | 1607 | 2605 | 1217 | 1432 | 2612 | 785 |
| Mortality Rate | 1176 (9.85) | 217 (23.51) | 385 (10.89) | 233 (19.38) | 416 (20.86) | 298 (13.19) | 499 (16.11) | 233 (25.98) | 326 (5.83) | 810 (13.69) | 267 (16.61) | 445 (17.08) | 181 (14.87) | 367 (25.63) | 443 (16.96) | 125 (15.92) |
| Male | 6646 (55.67) | 522 (56.55) | 1918 (54.26) | 651 (54.16) | 1063 (53.31) | 1255 (55.56) | 1657 (53.49) | 531 (59.2) | 3092 (55.33) | 3366 (56.9) | 871 (54.2) | 1508 (57.89) | 654 (53.74) | 790 (55.17) | 1378 (52.76) | 455 (57.96) |
| *Birth weight* |  |  |  |  |  |  |  |  |  |  |  |  |  |  |  |  |
| 1000 grams & Below (ELBW) | 131 (1.1) | 34 (3.68) | 59 (1.67) | 56 (4.66) | 85 (4.26) | 68 (3.01) | 47 (1.52) | 25 (2.79) | 93 (1.66) | 173 (2.92) | 67 (4.17) | 92 (3.53) | 42 (3.45) | 53 (3.7) | 81 (3.1) | 12 (1.53) |
| 1001 - 1499 grams (VLBW) | 504 (4.22) | 152 (16.47) | 147 (4.16) | 169 (14.06) | 216 (10.83) | 162 (7.17) | 246 (7.94) | 140 (15.61) | 241 (4.31) | 452 (7.64) | 180 (11.2) | 350 (13.44) | 131 (10.76) | 183 (12.78) | 224 (8.58) | 76 (9.68) |
| 1500 - 2499 grams (LBW) | 2910 (24.38) | 275 (29.79) | 643 (18.19) | 447 (37.19) | 598 (29.99) | 610 (27) | 913 (29.47) | 306 (34.11) | 1121 (20.06) | 1590 (26.88) | 556 (34.6) | 773 (29.67) | 332 (27.28) | 511 (35.68) | 765 (29.29) | 277 (35.29) |
| 2500 - 4000 grams (NBW) | 7889 (66.08) | 434 (47.02) | 2559 (72.39) | 496 (41.26) | 848 (42.53) | 1317 (58.3) | 1764 (56.94) | 405 (45.15) | 3823 (68.41) | 3072 (51.93) | 753 (46.86) | 1210 (46.45) | 647 (53.16) | 633 (44.2) | 1371 (52.49) | 405 (51.59) |
| > 4000 grams (Macrosomia) | 504 (4.22) | 28 (3.03) | 127 (3.59) | 34 (2.83) | 247 (12.39) | 102 (4.52) | 128 (4.13) | 21 (2.34) | 310 (5.55) | 629 (10.63) | 51 (3.17) | 180 (6.91) | 65 (5.34) | 52 (3.63) | 171 (6.55) | 15 (1.91) |
|  |  |  |  |  |  |  |  |  |  |  |  |  |  |  |  |  |
| Intravenous antibiotics | 5743 (48.11) | 315 (34.13) | 2148 (60.76) | 741 (61.65) | 256 (12.84) | 987 (43.69) | 1892 (61.07) | 280 (31.22) | 1330 (23.8) | 3015 (50.96) | 1176 (73.18) | 1966 (75.47) | 251 (20.62) | 382 (26.68) | 1051 (40.24) | 638 (81.27) |
| Intravenous fluids | 4158 (34.83) | 315 (34.13) | 2651 (74.99) | 743 (61.81) | 739 (37.06) | 844 (37.36) | 1278 (41.25) | 692 (77.15) | 1740 (31.14) | 3930 (66.43) | 1131 (70.38) | 1929 (74.05) | 533 (43.8) | 628 (43.85) | 1543 (59.07) | 609 (77.58) |
| Enteral feeds | 3485 (29.19) | 6 (0.65) | 31 (0.88) | 91 (7.57) | 240 (12.04) | 230 (10.18) | 147 (4.74) | 53 (5.91) | 2658 (47.57) | 993 (16.78) | 55 (3.42) | 19 (0.73) | 185 (15.2) | 21 (1.47) | 228 (8.73) | 11 (1.4) |
| Oxygen | 1776 (14.88) | 98 (10.62) | 1213 (34.31) | 572 (47.59) | 272 (13.64) | 913 (40.42) | 251 (8.1) | 257 (28.65) | 1277 (22.85) | 552 (9.33) | 190 (11.82) | 278 (10.67) | 94 (7.72) | 335 (23.39) | 175 (6.7) | 172 (21.91) |
| Phenobarbital | 814 (6.82) | 142 (15.38) | 213 (6.03) | 174 (14.48) | 202 (10.13) | 206 (9.12) | 273 (8.81) | 100 (11.15) | 398 (7.12) | 317 (5.36) | 193 (12.01) | 217 (8.33) | 95 (7.81) | 188 (13.13) | 214 (8.19) | 157 (20) |
| *Hospital used in the SENSS and NETS model derivation and temporal validation stage.  ^ǂ^Observations missing data on the outcome variable or all the candidate predictor variables omitted from analysis for objective two | | | | | | | | | | | | | | | | |

| Table S7: Distribution of predictors and missingness in SENSS external validation dataset by in-hospital mortality | | | |
| --- | --- | --- | --- |
| Predictors | **Present, n (%)** | **Missing, n (%)** | **Died, n (%)** |
| Difficulty feeding | 7741 (14.36) | 7248 (13.44) | 2269 (29.31) |
| Convulsions | 1975 (3.66) | 6845 (12.7) | 551 (27.9) |
| Central cyanosis | 1950 (3.62) | 3013 (5.59) | 762 (39.08) |
| Severe Indrawing | 3112 (5.77) | 5299 (9.83) | 1407 (45.21) |
| Floppy/inability to suck | 17389 (32.26) | 7886 (14.63) | 4485 (25.79) |
| None | 23559 (43.7) |  | 965 (4.1) |

| Table S8: Distribution of predictors in NETS external validation dataset by in-hospital mortality | | |
| --- | --- | --- |
| Predictors | **Prescribed, n (%)** | **Died, n (%)** |
| Intravenous antibiotics | 21125 (46.85) | 3541 (16.76) |
| Intravenous fluids | 19651 (43.58) | 3699 (18.82) |
| Enteral feeds | 6053 (13.42) | 279 (4.61) |
| Oxygen | 7700 (17.08) | 1830 (23.77) |
| Phenobarbital | 4103 (9.1) | 1116 (27.2) |
| None | 8168 (18.11) | 828 (10.14) |
