# Supplementary material for: External validation of inpatient neonatal mortality prediction models in high-mortality settings
Source: BMC Med. 2022 Aug 3;20:236. doi: 10.1186/s12916-022-02439-5 (PMC9347100; doi:10.1186/s12916-022-02439-5)
Supplement: Supplementary file 2 — Additional file 2. Model recalibration approaches evaluated. [file 12916_2022_2439_MOESM2_ESM.docx]

## **Additional file 2**

### **Model recalibration approaches evaluated**

Additional file 2: Figure S1 illustrates our attempt at logistic recalibration approach using the model updating dataset before model revision was considered. Model recalibration approaches like logistic recalibration are explained in detail elsewhere [21, 32]. We explored logistic recalibration because intercept recalibration would not suffice given the poor performance of both calibration slope and intercept from the original SENSS and NETS models (Figure 2). In logistic recalibration, intercepts and coefficients for corresponding linear predictors are estimated in order to update the regression coefficients [21, 32]. This method is meant to correct miscalibration of the predicted probabilities from the original SENSS and NETS and SENSS model (Figure 2), such that there is no general over- or underestimation of risks and such that predicted risks are on average not overly extreme or overly modest [21, 32].


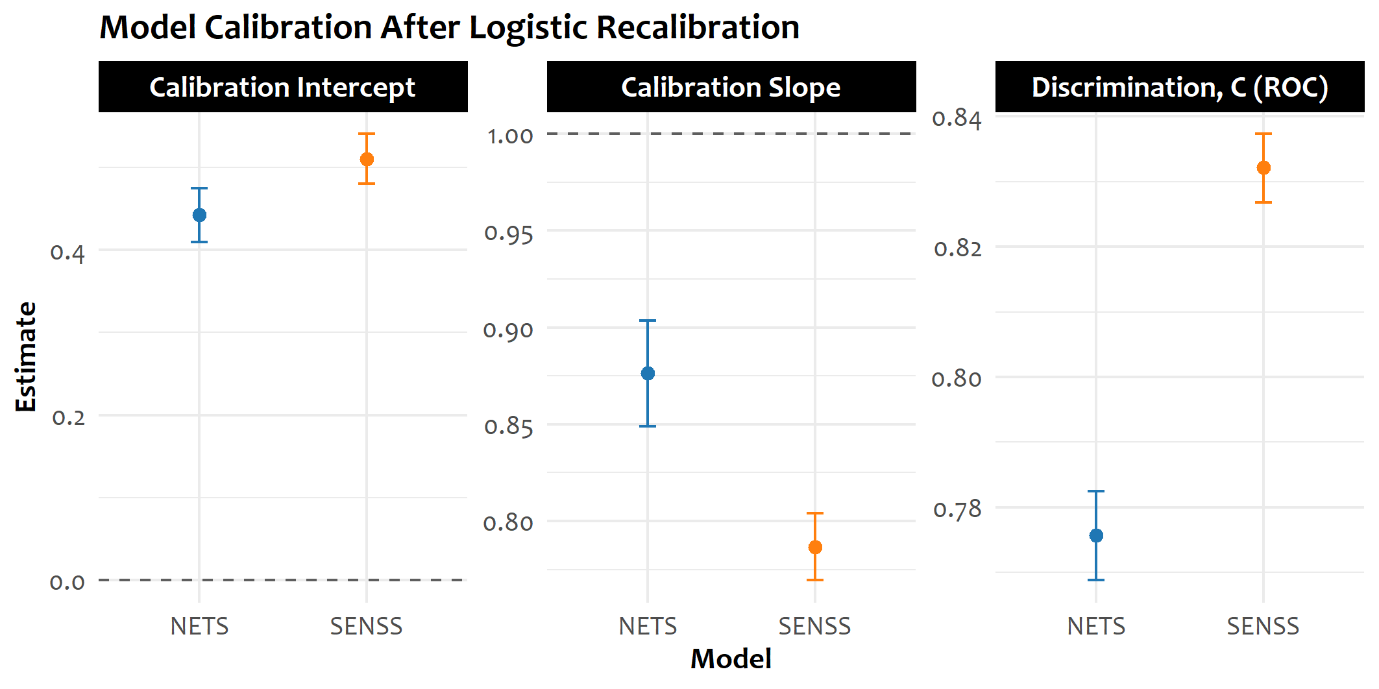


Figure S1: Model logistic recalibration results. SENSS: Score for Essential Neonatal Symptoms and Signs; NETS: Neonatal Essential Treatment Score.

While the logistic recalibration is one of the simpler approaches to model updating, it works best when the new dataset has a case-mix that is similar in both the updating and the external validation datasets [21]; This does not appear to be the case in our study given the differing levels of the predictor and outcome variables across the derivation, temporal validation, updating and external datasets (Table1, Additional file 1: Table S4 and Table S5) and as illustrated by Additional file 2: Figure S1. The level of the NETS model discrimination is also concerning (Figure 2), and the strength of the association between some predictors and the outcome could arguably be substantially different in the external validation population given the differences in the presence of some predictors and the outcome rate between the derivation, updating, and external validation datasets (Table1, Additional file 1: Table S4 and Table S5).

Logistic recalibration of SENSS and NETS models illustrated by Additional file 2: Figure S1 used the parameters in equation 1 and 2 for SENSS and NETS models respectively. The recalibrating dataset (SENSS: n=8848, NETS: n=6610), was from the original derivation hospital but was not included in the model development or internal validation and is represented by the *Model updating* column in Additional file 1: Table S4 and Table S5.

${LP}_{SENSS \left( recalibrated \right)}= \alpha_{1}+ \beta_{1} \times{LP}_{SENSS \left( original \right)} ( SEQ Eq \backslash* MERGEFORMAT 1)$

*Where*: $\alpha_{1}= -1.3884, \beta_{1}=0.7179$

$${LP}_{NETS \left( recalibrated \right)}= \alpha_{2}+ \beta_{2} \times{LP}_{NETS \left( original \right)} ( SEQ Eq \backslash* MERGEFORMAT 2)$$

*Where*: $\alpha_{2}= -1.6255, \beta_{2}=0.5316$
